# Supplementary figures and images for: High expression of Tie-2 predicts poor prognosis in primary high grade serous ovarian cancer
Source: PLoS One. 2020 Nov 5;15(11):e0241484. doi: 10.1371/journal.pone.0241484 (PMC7644024; doi:10.1371/journal.pone.0241484)

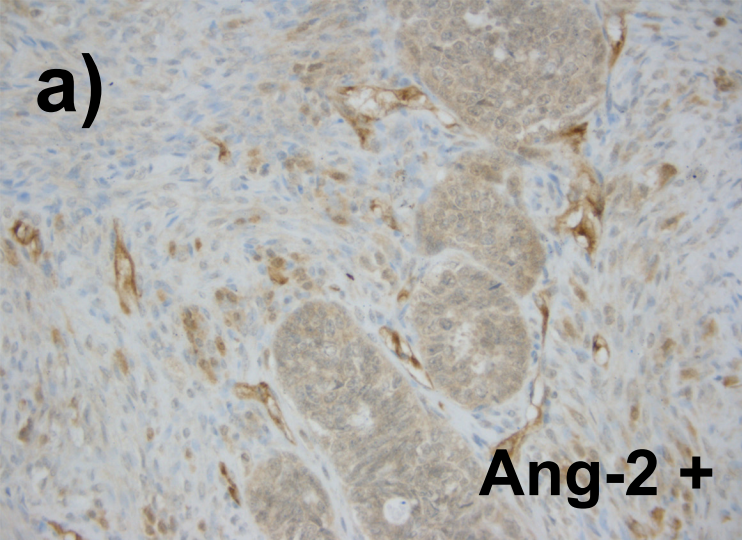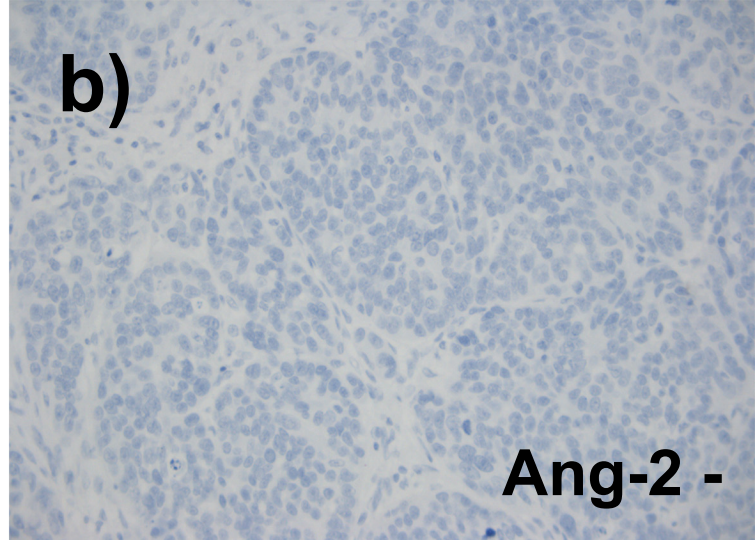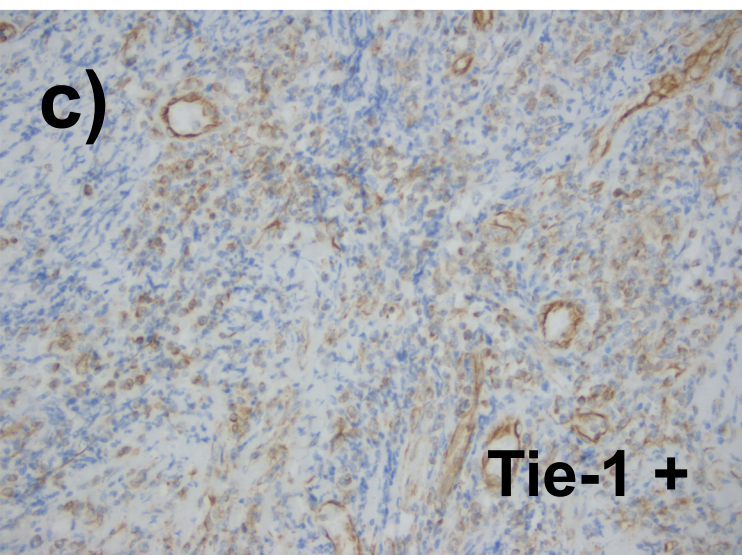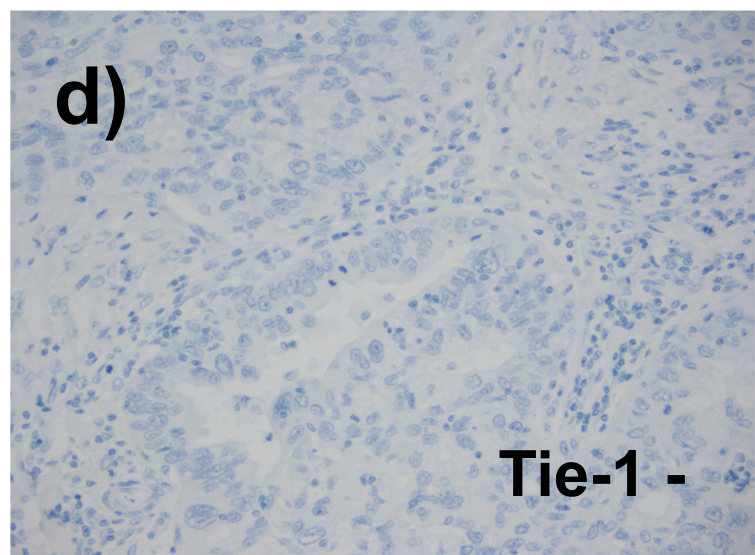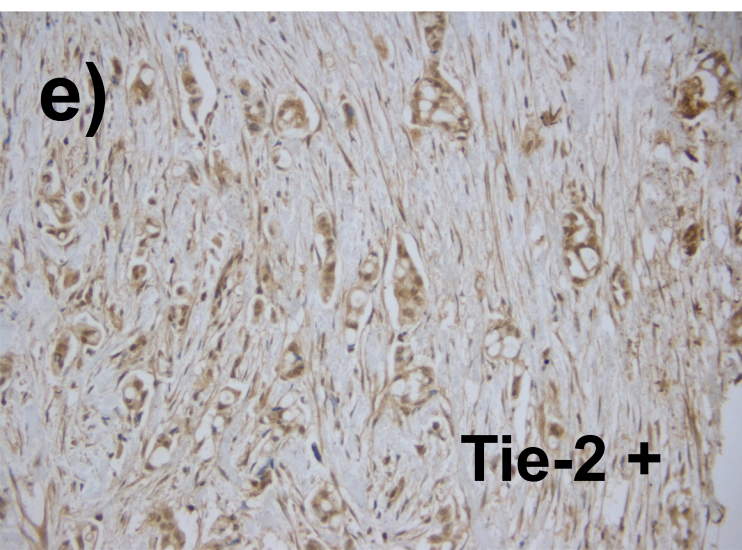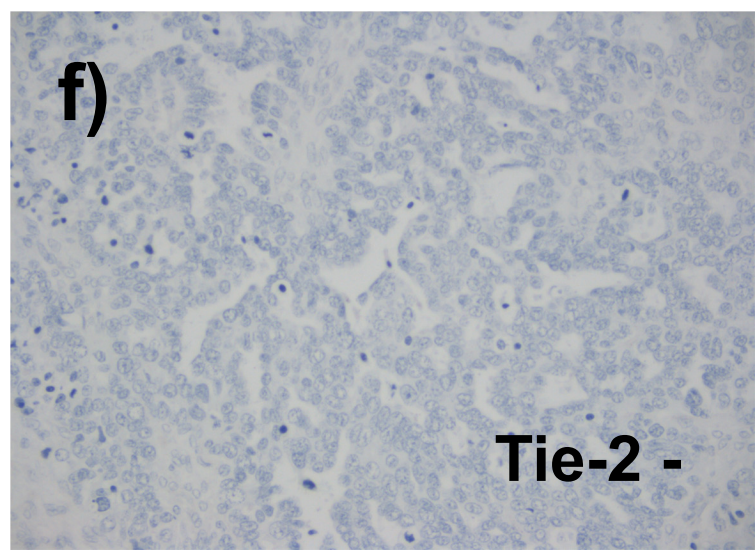

Supplement: S1 Fig — Positive and negative tissue control stainings for a) Ang-2 positive in ovarian tissue, c) Tie-1 positive in tonsillar tissue, e) Tie-2 positive in ovarian carcinoma, b), d) and f) are corresponding Ang-2, Tie-1 and Tie-2 ovarian carcinoma negative control stainings (primary antibody omitted). (PDF) [file pone.0241484.s001.pdf]
