# Supplementary material for: High expression of Tie-2 predicts poor prognosis in primary high grade serous ovarian cancer
Source: PLoS One. 2020 Nov 5;15(11):e0241484. doi: 10.1371/journal.pone.0241484 (PMC7644024; doi:10.1371/journal.pone.0241484)
Supplement: S1 File — (DOCX) [file pone.0241484.s003.docx]

**S1** **Table**

| **Variable** | **Tumor sample** | **Plasma/ serum** | **RNA** |
| --- | --- | --- | --- |
| **Ang-2** | Zhang L., 2003 [1]:  52 specimen  **Result**: Expression in endothelium and 12% of tumor cells | Sallinen H., 2010 [5]:  150 patients  **Result:** Higher pre-operative Ang-2 levels in cancer. Poor PFS and OS. | Hata K., 2004 [9]:  85 specimens  **Result**: Low Ang-1/ Ang-2 predicted poor OS (univariate analysis) |
|  | Lin Z., 2011 [2]:  21 patients  **Result:** No correlation with clinicopathological parameters, no difference between cancer vs benign | Sallinen H., 2014 [6]:  75 patients  **Result:** Higher Ang-2 and Ang-2/ VEGFR2 in cancer. Poor PFS and OS. |  |
| **Tie-2/ Ang-2** | Li S., 2010 [3]:  12 specimens  **Result:** Cytoplasmic expression, higher expression in cancer, no correlation with clinical parameters | Backen A.,  Clin Cancer Res 2014 [7]**:**  91 patients  **Result:** High Ang-1/Tie-2 improved PFS in bevacizumab treated patients | Hata K.,  Oncology 2002 [10]**:**  19 patients  **Result:** No difference with Ang-2 and Tie-2 expression in cancer vs benign tumors |
|  | Brunckhorst M.K., 2014 [4]:  12 cancer and 12 omental metastatic samples  **Result:** Ang-2 expression in tumor cells, Tie-2 in endothelium and fibroblasts. Higher Ang-2 expression in cancer. | Zhou C., 2016 [8]:  92 patients  **Result:** 50% increase of Tie-2 from the nadir predicted tumor progression after bevacizumab |  |

**S2 Table**

| **Variable** | **Correlation coefficient**  **(r)** | ***p*** |
| --- | --- | --- |
|  |  |  |
| **Ang-2 IRS** | -0.64 | **0.002** |
| **Ang-2 IRS*** | -0.79 | **0.004** |
| **Ang-2 IRS**  **(HGSC)** | -0.87 | **<0.001** |
| **Ang-2 met**  **PP** | 0.75 | **0.005** |
| **Tie-1 IRS** | 0.008 | 0.972 |
| **Tie-1 IRS**  **(*/HGSC)** | -0.087 | 0.799 |
| **Tie-1 met**  **IRS** | 0.215 | 0.502 |
| **Tie-2 IRS** | 0.163 | 0.479 |
| **Tie-2 IRS**  **(*/HGSC)** | -0.319 | 0.339 |
| **Tie-2 met IRS** | 0.211 | 0.510 |

p-values determined by Spearman`s test, IRS immunoreactive score,

HGSC high grade serous carcinoma, Ang-2 met angiopoietin-2 metastasis, PP

percentage of positively stained epithelial cells, * serous histologic type

**S3 Table**

|  | **Ang2** | ***p*** | **Tie1** | ***p*** | **Tie2** | ***p*** |
| --- | --- | --- | --- | --- | --- | --- |
| **Grade** |  |  |  |  |  |  |
| *Low* |  |  |  |  |  |  |
| IRS | 8 |  | 3 |  | 2 |  |
| PP | 80 |  | 65 |  | 40 |  |
| *High* |  |  |  |  |  |  |
| IRS | 6 | 0.075 | 2 | **0.018** | 2 | 0.108 |
| PP | 65 | **0.008** | 25 | **0.007** | 30 | **0.016** |
| **Stage** |  |  |  |  |  |  |
| *I-II* |  |  |  |  |  |  |
| IRS | 9 |  | 2 |  | 2 |  |
| PP | 75 |  | 20 |  | 40 |  |
| *III-IV* |  |  |  |  |  |  |
| IRS | 6 | 0.185 (**0.047***) | 2 | 0.781 | 2 | 0.141 |
| PP | 65 | 0.370 | 35 | 0.657 | 35 | 0.375 |
| **Histology** |  |  |  |  |  |  |
| *Serous* |  |  |  |  |  |  |
| IRS | 6 | 0.436 | 2 | **0.002** | 2 | **0.002** |
| PP | 65 | 0.110 | 40 | **0.002** | 30 | **<0.001** |
| *Mucinous* |  |  |  |  |  |  |
| IRS | 6 | 0.867 | 3 | **0.029** | 6 | **0.001** |
| PP | 75 | 0.255 | 60 | **0.024** | 75 | **<0.001** |
| *Endometrioid* |  |  |  |  |  |  |
| IRS | 6 | 0.175 | 2 | 0.411 | 2 | 0.672 |
| PP | 65 | 0.878 | 20 | 0.136 | 33 | 0.890 |
| *Clear cell* |  |  |  |  |  |  |
| IRS | 4 | 0.477 | 1 | **0.001** | 1 | 0.095 |
| PP | 30 | **0.020** | 5 | **0.003** | 10 | **0.024** |
| **Residual tumor** |  |  |  |  |  |  |
| *None* |  |  |  |  |  |  |
| IRS | 6 |  | 2.5 |  | 3 |  |
| PP | 70 |  | 45 |  | 40 |  |
| *≤1cm* |  |  |  |  |  |  |
| IRS | 6 | 0.109 | 2.5 | 0.583 | 2 | 0.923 |
| PP | 65 | 0.746 | 45 | 0.496 | 30 | 0.854 |
| *>1cm* |  |  |  |  |  |  |
| IRS | 6 | **0.012** | 2 | 0.465 (**0.008***) | 2 | 0.688 |
| PP | 60 | 0.092 | 25 | 0.284 (**0.020***) | 30 | 0.526 |
| **Recurrence** |  |  |  |  |  |  |
| *No* |  |  |  |  |  |  |
| IRS | 6 |  | 3 |  | 3 |  |
| PP | 73 |  | 45 |  | 42.5 |  |
| *Yes* |  |  |  |  |  |  |
| IRS | 6 | **0.018** | 2 | 0.157 (**0.025***) | 2 | 0.202 |
| PP | 63 | **0.006** | 25 | 0.091 (**0.009***) | 30 | 0.137 |

p values determined by Kruskal-Wallis test followed by Mann-Witney tests when appropriate; *p value reported for serous tumors in brackets when only comparison for this subgroup was statistically significant; IRS, PP are median values; HGSC = high grade serous cancer, IRS immunoreactive score, PP percentage of positively stained cells, Recurrence = recurrence of cancer

**References**

1. Zhang L, Yang N, Park J, Katsaros D, Fracchioli S, Cao G, et al. Tumor-derived Vascular Endothelial Growth Factor Up-Regulates Angiopoietin-2 in Host Endothelium and Destabilizes Host Vasculature, Supporting Angiogenesis in Ovarian Cancer. Cancer Research. 2003;63: 3403-3412.

2. Lin Z, Liu Y, Sun Y, He X. Expression of Ets-1, Ang-2 and maspin in ovarian cancer and their role in tumor angiogenesis. Journal of experimental & clinical cancer research : CR. 2011;30: 31. doi: 10.1186/1756-9966-30-31.

3. Li S, Meng L, Zhu C, Wu L, Bai X, Wei J, et al. The universal overexpression of a cancer testis antigen hiwi is associated with cancer angiogenesis. Oncology reports. 2010;23: 1063. doi: 10.3892/or_00000733.

4. Brunckhorst MK, Xu Y, Lu R, Yu Q. Angiopoietins Promote Ovarian Cancer Progression by Establishing a Procancer Microenvironment. American Journal of Pathology, The. 2014;184: 2285-2296. doi: 10.1016/j.ajpath.2014.05.006.

5. Sallinen H, Heikura T, Laidinen S, Kosma VM, Heinonen S, Ylä-Herttuala S, et al. Preoperative Angiopoietin-2 Serum Levels: A Marker of Malignant Potential in Ovarian Neoplasms and Poor Prognosis in Epithelial Ovarian Cancer. International Journal of Gynecological Cancer. 2010;20: 1498-1505. doi: 10.1111/IGC.0b013e3181f936e3.

6. Sallinen H, Heikura T, Koponen J, Kosma VM, Heinonen S, Ylä-Herttuala S, et al. Serum angiopoietin-2 and soluble VEGFR-2 levels predict malignancy of ovarian neoplasm and poor prognosis in epithelial ovarian cancer. BMC Cancer. 2014;14: 696. doi: 10.1186/1471-2407-14-696.

7. Backen A, Renehan AG, Clamp AR, Berzuini C, Zhou C, Oza A, et al. The combination of circulating Ang-1 and Tie-2 levels predicts progression-free survival advantage in bevacizumab treated patients with ovarian cancer. Clin Cancer Res. 2014.

8. Zhou C, Clamp A, Backen A, Berzuini C, Renehan A, Banks RE, et al. Systematic analysis of circulating soluble angiogenesis-associated proteins in ICON7 identifies Tie2 as a biomarker of vascular progression on bevacizumab. The British Journal of Cancer. 2016;115: 228-235. doi: 10.1038/bjc.2016.194.

9. Hata K, Nakayama K, Fujiwaki R, Katabuchi H, Okamura H, Miyazaki K. Expression of the angopoietin-1, angopoietin-2, Tie2, and vascular endothelial growth factor gene in epithelial ovarian cancer. Gynecologic Oncology. 2004;93: 215-222. doi: 10.1016/j.ygyno.2003.12.031.

10. Hata K, Udagawa J, Fujiwaki R, Nakayama K, Otani H, Miyazaki K. Expression of Angiopoietin-1, Angiopoietin-2, and Tie2 Genes in Normal Ovary with Corpus luteum and in Ovarian Cancer. Oncology. 2002;62: 340-348. doi: 10.1159/000065066.
