# Supplementary material for: High expression of Tie-2 predicts poor prognosis in primary high grade serous ovarian cancer
Source: PLoS One. 2020 Nov 5;15(11):e0241484. doi: 10.1371/journal.pone.0241484 (PMC7644024; doi:10.1371/journal.pone.0241484)
Supplement: Attachment — Submitted filename: Response to Reviewers.docx [file pone.0241484.s004.docx]

**Response to Academic Editor**

We thank Academic Editor for the valuable comments. Each of your comment or question is indicated in bold followed by our response. All changes are also highlighted in the “Revised manuscript with track changes” and corrected in the unmarked version of revised manuscript without track changes.

1. **Specifically, the authors should include controls, improve quality of figures and consistency of the presentation of IHC data, details of IHC and editorial corrections including sentences.**

We included 30 IHC control tissue samples in this study: 22 positive and 8 negative controls of human tissues (adnexal, uterine and tonsillar samples) positive for studied markers.

(added in Materials and Methods, Immunohistochemistry, page 8, lines 151-55)

We improved the quality of figures. Figure 2 (Figure 3 in the first version of the manuscript) is made newly in order to clear the image. Former Figure 2 was removed. Also Figures 1 and 3 were made newly to improve the consistency of the presentation and clarify the IHC data.

We removed the positive percentage (PP) and staining intensity (SI) parameters from the manuscript, tables (2 and 3, page 14-16, lines 299-307) and the figures (1 and 3) and used only immunoreactive score (IRS) data for the semi-quantitative analysis to improve the presentation of the details of IHC data. We left the parameters in the supplementary table 2 and 3 to clarify which parameters explain the result.

The English language and editorial corrections for this manuscript was made by Dr Ewen MacDonald.

2. **To enhance the reproducibility of your results, we recommend that if applicable you deposit your laboratory protocols in protocols.io, where a protocol can be assigned its own identifier (DOI) such that it can be cited independently in the future.**

Our laboratory protocols are the standard, official, qualified protocols used in the Department of Pathology at the University of Eastern Finland. The protocols are openly delivered, but not available in English at this point. So we will work on that and will deposit the protocols in protocols.iu in future.

3. **Please ensure that your manuscript meets PLOS ONE's style requirements, including those for file naming.**

We have ensured that the manuscript meets PLOS ONE`s style requirements, including file naming.

4. **Please provide additional details regarding participant consent. In the ethics statement in the Methods and online submission information, please ensure that you have specified (1) whether consent was informed and (2) what type you obtained (for instance, written or verbal, and if verbal, how it was documented and witnessed). If your study included minors, state whether you obtained consent from parents or guardians. If the need for consent was waived, please ensure that you have discussed whether all data were fully anonymized before you accessed them and/or whether the IRB or ethics committee waived the requirement for informed consent”.**

The written, informed consent was obtained from the patients participating in this study. Methods page 8, lines 143-44.

5. **In your Methods section, please provide additional information about the participant recruitment method and the demographic details of your participants. Please ensure you have provided sufficient details to replicate the analyses such as: a) the recruitment date range (month and year), b) a table of relevant demographic details and c) a description of how participants were recruited.**

All patients were Finnish, white females (Caucasian), median age 58 years (26-88). They were recruited in the gynecological policlinic, when they were sent for the diagnostics and treatment of ovarian tumors. Recruitment date range was 1.9.1999 ⎯28.3.2007.

Methods page 5, lines 113, 123-24.

6. **We suggest you thoroughly copyedit your manuscript for language usage, spelling, and grammar. If you do not know anyone who can help you do this, you may wish to consider employing a professional scientific editing service.**

The English language corrections and copyediting for this manuscript was made by Dr Ewen MacDonald.

A point-by-point Response letter to the Reviewers

**Response to Reviewer 1**

We thank Reviewer for the valuable comments. Each of your comment or question is indicated in bold followed by our response. All changes are also highlighted in the “Revised manuscript with track changes” and corrected in the unmarked version of revised manuscript without track changes.

1. **Immunohistology is the primary method used to monitor the expression of the angiogenesis markers in ovarian tissues. Therefore, it is important that the IHC data be of high quality. The expression of Tie-2 is diffuse and in some of the images provided by the authors, it is not clear if the expression is sufficiently specific. Authors are requested to demonstrate antibody specificity. At the same time, this study will also benefit from the inclusion of positive controls of human tissues that are known to be positive for Tie-2, Ang-2 and the other markers used in this study.**

Immunohistochemical stainings have been implemented according to the official protocols by the Department of Pathology at the University of Eastern Finland by using the antibodies of Ang-2 by Novus, Tie-1 by Abcam and Tie-2 by Santa Cruz as described in the Materials and Methods Immunohistochemistry section (page 8, lines 147-51).

The antibodies used have been checked by the vendors and the Western blots have been made.

In addition to confirm the expression of growth factors and receptors we performed qRT-PCR

to see their expression in the RNA-level.

We included 30 IHC control tissue samples in this study: 22 positive and 8 negative controls of human tissues (adnexal, uterine and tonsillar samples) positive for aforementioned markers.

(added in Materials and Methods, Immunohistochemistry, page 8, lines 151-55)

Figure 2 (Figure 3 in the first version of the manuscript) is made newly in order to clear the image. Former Figure 2 was removed.

2. **Authors have used the immunoreactivity score (IRS) which takes into account the percent of positive cells and the intensity of the staining. It is therefore confusing as to why the authors selectively show IRS data in some figures and positive percentage (PP) and specific intensity (SI) for others. It will be best if authors use IRS data for the semi-quantitative analysis. Alternatively, authors may also conduct the quantitation using the accepted H-scoring system.**

We removed the positive percentage (PP) and staining intensity (SI) parameters from the manuscript, tables (2 and 3, page 14-16, lines 299-307) and the figures (1 and 3) and used only immunoreactive score (IRS) data for the semi-quantitative analysis. We left the parameters in the supplementary table 2 and 3 to clarify which parameters explain the result.

We did not use H-score, because it is used to evaluate the nuclear positivity.

3. **There is no correlation between IHC expression and mRNA expression. Authors point out that this is likely because the RNA samples are derived from the entire tissue and not from cancer, endothelial or stromal cells. This logic makes sense. But it also implies that there is no necessity to include the qPCR results as they only confuse the results.**

We removed the qRT PCR results from the manuscript. We would like to leave it in the supplementary material (S4 Table), because it is considered the other base result of the study.

4. **Authors should specify if the samples used for IHC were obtained from treatment naïve patients.**

Added in Materials and Methods, Patients and data collection, page 5, line 114-15.

5. **Overall, the results are presented in a confusing manner. Often times, in the results section, the number of the figure or the table is not explicitly mentioned. Therefore, it is hard for the reader to determine which data the authors are referring to in the text.**

We defined the presentation of the results, specified and added the references to the figures and tables.

**Response to Reviewer 2**

We thank Reviewer for the valuable comments. Each of your comment or question is indicated in bold followed by our response. All changes are also highlighted in the “Revised manuscript with track changes” and corrected in the unmarked version of revised manuscript without track changes.

1. **Several sentences were incomplete in the manuscript, so extensive language correction is required.**

English language correction has been made for this manuscript in January 2020 by Dr Ewen MacDonald.

2. **There is no clear result given the angiopoietin-2 and Tie-2 in other subtypes shown in sample numbers.**

The number of other subtypes than high grade serous and endometrioid carcinoma is very small (mucinous tumors 11 and clear cell 5, others 4), so making a definitive conclusions or giving exact statistical results of small subgroups is not relevant.

3. **The expression of angiopoietin-2 and Tie-2 in distal omental metastasis is showing an interesting result; however, the discussion is missing.**

We have discussed about the significance of the higher expression of the metastases in Discussion page 18-19, lines 373-85.

4. **Since it is correlated with the omental metastasis, what is the status in other metastasis?**

We included only omental metastases in this study, since this type of dissemination is typical for the high grade ovarian cancer. To evaluate the other metastases, could be the idea for future studies.

5. **There is no statistical calculation shown in qRT-PCR of all the molecules. How many mRNA samples were used for this analysis, and how many times, experiments were repeated to calculate significance? Because this is the base result for the manuscript.**

Samples from 22 primary and 15 metastatic tumors were analysed by qRT-PCR for each antibody (total 66 primary and 45 metastatic samples). Each sample has been repeated three times to calculate the significance. (Materials and Methods, qRT-PCR, page 9, line 174)

Only the significant correlations between the immunohistochemistry and the mRNA levels are shown in the Supplementary table 4.

6. **There is no H-score calculated for the intensity of Tie-1, two, and Ang 2 staining in serous ovarian cancer samples. What is the subcellular localization of these molecules?**

We did not use H-score, because it is used to evaluate the nuclear positivity. Ang-2 expression was detected mainly in the cytoplasm of the tumor epithelial cells and vascular endothelial cells. Tie-2 stained most in the cytoplasm and the membranes of the tumor epithelial cells, vascular endothelium and particularly in metastases stromal fibroblasts. Tie-1 expression was practically not seen in the tumor epithelial cells, it was strongest in the tumor stromal cells. (Results pages 10-11, lines 202-08, 222-33)

7. **Immunohistochemistry images are showing poor quality of staining.**

Figure 2 (Figure 3 in the first version of the manuscript) is made newly in order to clear the image. Former Figure 2 was removed.
