# Supplementary material for: High expression of Tie-2 predicts poor prognosis in primary high grade serous ovarian cancer
Source: PLoS One. 2020 Nov 5;15(11):e0241484. doi: 10.1371/journal.pone.0241484 (PMC7644024; doi:10.1371/journal.pone.0241484)
Supplement: Attachment — Submitted filename: Response to Reviewers2.docx [file pone.0241484.s005.docx]

A point-by-point Response letter to the Reviewers

**Response to Reviewer 3**

We thank Reviewer for the valuable comments. Each of your comment or question is indicated in bold followed by our response. All changes are also highlighted in the “Revised manuscript with track changes” and corrected in the unmarked version of revised manuscript without track changes.

1. **The manuscript thematically purports to investigate biomarkers of tumour angiogenesis in ovarian cancer. Ang-2 and Tie-2 protein levels assessed by immunohistochemistry are shown to relate to overall survival; however the predominant expression of both of these proteins is in epithelial tumour cells. Although the predictive value of these markers appears to be strong, it is unclear how this relates to tumour angiogenesis - microvessel density is not assessed in this study.** Furthermore**, while the Discussion maintains the focus on Ang2/Tie2 in tumour angiogenesis and metastasis, it appears to ignore studies in other tumour types showing that Ang2 signaling through integrins on tumour cells can cell-intrinsically promote invasion and metastasis by inducing epithelial to mesenchymal transition (EMT; e.g. Imanishi et al. 2007 Cancer Research 67(9):4254–63; Dong et al. 2018 Oncotarget 9:12705-12717, and others). This alone could explain the observation of higher Ang2 (and possibly Tie2) expression in metastases than in primary tumours. The authors should acknowledge promotion of EMT by Ang2 as a possible mechanism, and acknowledge the limitations of their data in making conclusions regarding tumour angiogenesis and systemic metastasis.**

Added the acknowledgement of promotion of EMT by Ang-2 as a possible mechanism of its higher expression in metastasis compared to the primary tumors and the limitation of this data.

Discussion, page 21, rows 401-406.

Added references 22 and 31. References, page 25, rows 525-27 and 26, rows 554-56 .

2. **In Figure 1 it is not clear what values are displayed – are these median values of IRS? Epithelial, stromal or overall? Presuming that an IRS value was calculated for each tumour specimen, and the data are non-normally distributed, the data could be better presented as a box-and-whisker plot (or similar) with individual data points. Currently there is no indication of the spread of the data.**

Figure 1 is now presented as a violin blot with individual data points, describing both the median values of IRS and the density of the distribution of the data points in each group.

IRS is calculated from the staining intensity and the percentage of positively stained cells of the tumor epithelial cells. Methods page 8, lines 159-66.

3. **Please provide images of the positive and negative tissue controls for Ang-2, Tie-2 and Tie-1 as Supporting Information, along with references indicating expected patterns of staining in positive control tissues for each protein. It is not sufficient merely to state that the negative controls were negative. Were antibody isotype controls included? If Western Blots have been performed to validate the specificity of the antibodies used for IHC, please present this data as well.**

Images of the positive and negative tissue controls for Ang-2, Tie-1 and Tie-2 are now included as Supporting Information (S1 Figure). References indicating expected patterns of staining in positive control tissues are also provided for each protein.

Methods page 8, lines 148-53; References 14-16, page 25.

Western blots were not included in this study. We believe that with immunohistochemical detection, we were able to detect the expression of growth factor and its receptors reliably, and analyse the exact location of these proteins in tumor tissue. The vendors of the antibodies have already checked and performed the Western blots for each antibody. In addition, to confirm the expression of growth factors and their receptors we performed qRT-PCR to see their expression in RNA level.

4. **Some of the data concerning Ang-2 expression are counterintuitive. Low Ang-2 expression is associated with poor overall survival in spite of higher Ang-2 immunoreactivity in metastases, and in contrast with studies in other tumour types showing that Ang-2 promotes tumour invasiveness (ref. 22). The negative correlation between Ang-2 immunoreactivity and mRNA levels is also perplexing. Although there are potential explanations for these discrepancies, it does raise suspicion about the specificity of the Ang-2 antibody used, and underscores the importance of independent validation of antibody specificity by investigators. Furthermore, the authors state in the Abstract that relative expression of Ang-2 determined by qRTPCR correlated significantly with IHC protein levels, which is misleading – the correlation is statistically significant, but negative.**

There are different results of the significance of Ang-2 expression on survival in different cancer types. Zhang L (ref.19) thought that Ang-2 is detectable in tumor cells in only 12% of tumor specimens of ovarian cancer. In contrast Brunckhorst (ref. 20) found that tumor cells commonly expressed Ang-2. In these earlier studies the correlation to the clinical factors has not been found in contrast to this study. In this study the low Ang-2 expression associated with poor overall survival is specifically the tumor epithelial cell expression in primary tumors! The metastases were not taken into count in this analysis.

In addition, low Ang-2 expression in primary tumors was associated with poor survival in univariate analysis, not in multivariate analysis. Other tissues have also been stained with these same antibodies with positive expression results in the laboratory of the Institute of Pathology in University of Eastern Finland.

The negative correlation of Ang-2 to the mRNA levels could be explained by the influence of the post-translational regulation of the protein synthesis and the distribution of Ang-2 protein in tumor samples. For the validation of the antibody used, please see Answer number 3, the second chapter.

Added negatively, in Abstract, page 2, line 57.

5. **Line 235: the authors state that “the correlation [between Ang-2 and Tie-1] was significant among other histological types”. If so, please present r and p values in the text or a table. Please clarify in the Methods section which statistical test was used to measure this correlation.**

Added r and p values in the text. Results, page 13, lines 246.

Correlations between immunohistochemical parameters were measured using the Spearman`s correlation test. Methods, page 10, lines 197-98.

6. **In Tables 2 and 3, the authors seem to only present data for analyses that yielded statistically significant results. Did all analyses performed yield statistically significant results? Please provide the results of all analyses for completeness. Were all parameters analysed for their relationship to overall survival also assessed regarding PFS? If so, please present these data as well.**

All analyses performed did not yield statistically significant results. All parameters analysed for their relationship to overall survival were also assessed regarding PFS.

Non-significant and PFS results are added to the Table 3 and Table 4 (former Table 2 and 3), pages 16-18.

7. **Similarly, if correlation analyses between IRS and qRTPCR results were performed for Tie1 and Tie2, please present these as well. Given that the authors have removed most of the qRTPCR data from the previous version of their manuscript, they should consider toning down claims of their study’s importance/uniqueness related to this.**

Correlation analyses between IRS and qRTPCR results were also performed for Tie-1 and Tie-2. These results are added to the Supplementary Table 3 (S3 Table).

In the first revision, we were suggested to remove the qRTPCR data. As a compromise, we left it to the Supplementary material, because on the other hand it was considered the base of the result.

8. **The authors state (line 97) that “there are no studies investigating the tissue expressions of Ang-2 and its receptors in ovarian cancer”, referencing Table S1, yet this Table contains several examples where such investigations appear to have been published, e.g. ref. 19. Please revise this statement.**

Revised the statement, ”there are only four studies with smaller populations and none investigating the tissue expression of Ang-2 and both its receptors in ovarian cancer”. Introduction, page 4, lines 97-98.

9. **Table S1: several references within this table do not appear within the main References list. Please add a list of full Supplementary references to the Supporting information file to cover these.**

List of full Supplementary references is added in Supplementary material.

10. **Statistical analysis: I am not familiar with the use of a Mann-Whitney U test as a post-hoc test for pairwise comparisons subsequent to the overarching Kruskal-Wallis test. In my understanding Dunn’s post-hoc test is more appropriate. Please provide a reference validating the appropriateness of this approach, or seek expert statistical advice.**

We have seeked expert statistical advice and according to the expert, the results are the same either by using the Mann-Whitney U test or Dunn`s post-hoc test. Both tests are appropriate and equal in this purpose, used as a post-hoc test for pairwise comparisons subsequent to the overarching Kruskal-Wallis test.

11. **Do the omental metastases examined reflect systemic or local/in transit metastasis?**

Omental metastases reflect local/ in transit intra-abdominal metastasis in ovarian cancer. It is the most typical intra-abdominal site for ovarian cancer to metastasize and omental metastasis over 2cm makes the ovarian cancer disseminated stage IIIc.

12. **S1 Table 1st row data, 3rd column: “Post-oper” – word incomplete.**

Corrected, Supplementary S1 Table, 1st row.

13. **Line 326: “angiogenetic” should be “angiogenic”**

Corrected, page 19, line 342.

**Response to Reviewer 4**

We thank Reviewer for the valuable comments. Each of your comment or question is indicated in bold followed by our response. All changes are also highlighted in the “Revised manuscript with track changes” and corrected in the unmarked version of revised manuscript without track changes.

1. **Figure 1 – Figure 1 and the Figure 1 legend both lack sufficient detail to interpret the immunohistochemistry data. Readers should be able to determine the distribution of staining scores across the cohort, which is not possible with data presented as a histogram. Please change histograms to violin plots, which will allow you to show the median score in each group, the individual values per patient, their distribution and the density. The figure or legend must also state the number of patients included in each group. A statistical test should also be used to compare expression values for each marker between primary tumors and metastases, and P values should be reported for each comparison. The y-axis also should be labelled. Please also state in the legend whether the IRS was calculated from the tumor epithelium or stroma.**

Figure 1 is now changed to violin plots. The y-axis is labelled as IRS and significant p-values are reported for each comparison in Figure 1.

The number of patients included in each group, statistical test and from which tissue/ compartment IRS was calculated were added to the Figure legend.

Results, page 10-11, lines 211-18.

2. **Results – Throughout the text the authors refer to staining patterns in primary high grade serous and endometrioid cancer. This data is not shown. For example, lines 205-206 – The authors report that “the stromal staining of Ang-2 was mostly weak in 74% of cases in primary high grade serous and endometrioid tumors”. No Figure or Table is referred to. When referring to staining intensity, perhaps the authors mean to refer to Table S2? However, Table S2 contains staining intensity for all histotypes combined. Where is the data presented for selected primary high grade serous and endometrioid tumors?**

To make the article more readable and not to repeat all the results, we decided to write the results of high grade serous and endometrioid cancer in the main text and the whole study population in the Table 2 (former Table S2). High grade serous cancer is the most common type of ovarian cancer and the results of that particular group are of the most interest.

Clarified in Results page 11, rows 237-38.

3. **Results, lines 224-225 – “stromal staining was strong in 64% of cases, emphasizing stromal fibroblasts and vascular endothelium (Figs 1 and 2c-d)”. Figures 1 and 2 do not show that 64% of cases have strong stromal staining. Authors need to carefully check that each results statement is correctly linked to the appropriate Figure and/or Table, and that the Figures and/or Tables referred to actually contain that data.**

Corrected the place of reference to the ”Figs 1 and 2c-d”.

Results, page 11, line 228.

Checked the references to the Figures and Tables.

4. **Table S2 – Given the amount of key data presented in Table S2, this should be changed to a main Table within the manuscript.**

Table S2 is relocated and changed to a main table within the manuscript, Table 2, pages 12-13.

5. **Table S2 – It is not clear whether the P values are calculated for just the paired primary and metastatic samples from the same patients, or unpaired groups of all primary and all metastases. Consider doing both. The range of IRS values are missing from the table.**

P values are calculated for the paired or related primary and metastatic samples from the same patients. That is clarified in the Table 2 (former S2 Table).

We do not want to mix all the different histological types and compare them to the metastasis of high grade serous tumors, as all the metastasis were high grade serous type. However there was not significant differencies to the results presented in the manuscript even when calculated with the unpaired groups.

The ranges of IRS are added to the Table 2 (former S2 Table).

6. **Results, lines 234-237 – “The level of Tie-1 expression was moderately, but significantly, correlated with its ligand Ang-2 (r = 0.5, p < 0.001) in primary serous ovarian tumors, and the correlation was also significant among other histological types. The expression level of Tie-2 showed a weak, but significant correlation with Ang-2 (r = 0.3, p = 0.012) and moderate correlation with Tie-1 (r = 0.5, p < 0.001) in primary cancer.” Which values were used to test these correlations and in which cellular compartment?**

IRS and PP values were used to test these correlations. IRS and PP are measured from the expressions of the tumor epithelial cells.

Clarified in Results page 13, lines 244-50.

7. **Results, lines 246-247 – “The level of Tie-1 expression did not differ between primary and metastatic lesions (Fig 1, 2 and S2 Table)”. However table S2 shows that the stromal expression of Tie-2 does differ between primary and metastatic lesions (p-value 0.046. Please clarify.**

Clarified in Results, page 14, lines 259-61.

8. **Table S3 – Please indicate whether the values were calculated from epithelial or stromal cells.**

The values were calculated from the tumor epithelial cells.

Added in Supporting information captions, page 28, lines 609-10.

9. **Results, lines 251-270 – Throughout this section the authors are investigating the association between biomarker expression and various clinical parameters. However, we are not shown the expression data. For example, “The low level epithelial expression of Ang-2 in primary tumors associated significantly with the ovarian cancer recurrence and with the greater residual tumor size (> 1cm) after primary surgery (p = 0.018, p = 0.012)”. For this comparison, the authors should show the median IRS for tumours with residual tumor > 1cm, compared to IRS of tumours with residual tumor < 1cm and no residual tumour. Therefore, in addition to the P values in Table S3, please include the median IRS and PP values for each group comparison.**

Table S2 (former Table S3) is made newly and now it shows median IRS an PP values for each group comparison. Checked by the statistician, in non-normally distributed groups, the median value can be the same even if there is a significant difference between the groups.

10. **Results, lines 254-256 – “In high grade serous tumors, high levels of Ang-2 staining were related to the resistance to platinum chemotherapy (p = 0.017) (S3 Table).” Platinum resistance and high-grade serous tumors are both not shown in Table S3.**

Platinum resistance and high grade serous cancer are mentioned only in the text, because other groups and the total study population had not significant results in relation to that parameter. S2 Table (former S3 Table) mainly consists of the results of the total study population although some results of the HGSC are shown for the interest of that particular group, as being the most common type of ovarian cancer.

11. **Results, line 284-286 – The authors need to explain how high Tie-2 is associated with shorter overall survival, and low Ang-2 is associated with poor survival, and yet Tie-2 and Ang-2 expression are positively correlated (as stated in Results lines 236-237). Presumably these are different populations of patients? Please explore and explain this result.**

IRS >2 of Tie-2 is associated with shorter overall survival of high grade serous ovarian cancer patients as indicated in Table 4. In turn IRS of Ang-2 ≤6 is related to poor survival when the whole study population is taken into count. This is clarified in Table 4, page 18.

When the whole study population is observed, Tie-2 and Ang-2 expressions are positively correlated. The other histologies in addition to high garde serous tumors make the correlation positive. In addition, it has to be noticed that the other one is the receptor while the other is the actual factor.

12. **Figure 3 – In the figure, please indicate the number of individuals at risk at each major time interval under each Kaplan-Meier plot.**

Indicated the number of individuals at risk at each major time intervals under each Kaplan-Meier plot in Figure 3.

13. **Figure 3 and Table 3 – How was high and low assigned for each biomarker, and what was the cut-off used for high and low?**

The median of the group was used for the cut-off for high and low for each biomarker.

14. **qRT-PCR is mentioned in the Abstract, the Methods and Discussion, however not mentioned in the Results. Please clarify.**

The results of qRT-PCR were relocated to the Supporting information (S3 Table) after previous revision. In the first revision, we were suggested to remove the qRTPCR data. As a compromise, we left it to the Supplementary material, because on the other hand it was considered the base of the result.

15. **Table S4 – Please include a table description indicating that Ang-2 IRS and PP is being correlated with qRT-PCR, and how the P values were calculated, and the numbers of samples tested. Also, please include the data for Tie-1 and Tie-2.**

Table description is included with the information mentioned above. Supporting information captions S3 Table (former S4 Table) page 28, lines 612-14.

qRT-PCR data for Tie-1 and Tie-2 and statistical test is included in S3 Table.

16. **Grammar needs to be corrected throughout the manuscript.**

Prof Ewen MacDonald corrected the english grammar of the manuscript.

17. **Table 3 – Table 3 shows 48 patients with low Ang-2 IRS and 82 patients with high Ang-2, which added together equals 130 patients. However, there are only 86 women described in the Materials and Methods. Please clarify. Also, Tie-1 is missing from this Table.**

Table 4 (former Table 3) and that particular column shows the 5 year survival percentage of each group mentioned, not the absolute number of patients. So, 48% of patients with low Ang-2 IRS are alive after five years of diagnosis and further, 82% of patients with high Ang-2 are alive after five years.

Tie-1 data is added to the Table 4, page 18.
