# Supplementary material for: High expression of Tie-2 predicts poor prognosis in primary high grade serous ovarian cancer
Source: PLoS One. 2020 Nov 5;15(11):e0241484. doi: 10.1371/journal.pone.0241484 (PMC7644024; doi:10.1371/journal.pone.0241484)
Supplement: Attachment — Submitted filename: A point-by-point response letter to the Reviewers.docx [file pone.0241484.s006.docx]

A point-by-point Response letter to the Reviewers

**Response to Reviewer 3**

We thank Reviewer for the valuable comments. Each of your comment or question is indicated in bold followed by our response. All changes are also highlighted in the “Revised manuscript with track changes” and corrected in the unmarked version of revised manuscript without track changes.

**Reviewer #3: The manuscript is overall improved, but there are some important points remaining to be addressed. Please see below, with numbers corresponding to the original questions raised. All points not specifically mentioned have been satisfactorily addressed.**

1. **The authors have acknowledged the potential role of Ang2 in EMT and metastasis, but have done so very superficially – in fact the statement I made regarding this in my initial review has been copied almost word for word into the manuscript, which is not acceptable. Please add your own insight. In asking for the authors to consider the limitations of the conclusions that can be drawn from their data re angiogenesis, I intended a more balanced assessment of the different possible mechanisms at play, rather than the blunt statement that has been inserted. Although the Discussion is overall appropriate, it remains unclear to me whether the observed relationship between tumour cell-expressed Tie2/Ang2 and survival/metastasis has anything to do with angiogenesis, and if so, how. A correlation analysis between tumour cell-expressed Tie2/Ang2 and blood vessel density would have been informative in this regard, but perhaps this must wait for a future study. See also point 11.**

We have added our insight of the potential role of Ang-2 in EMT and metastasis and also the assessement of the different possible mechanisms in metastasis in Discussion, page 22, rows 411-423.

In earlier studies, it has been concluded that tumor cells express and secrete several angiogenic factors including Tie2/Ang2 to the tumor microenvironment (for ex. Gavalas N, Int J Mol Sci 2013).

As reviewer mentions, the correlation of Tie-2/Ang-2 with blood vessel density will need further study.

2. **Original question 3. Thank you for adding the images in S1 Figure. Please add detail in the caption to describe what tissues are stained in each image, and what the negative control represents – negative control tissue, or negative control antibody?**

Details of the caption for S1 Figure added, page 29, rows 643-646.

A sample without a primary antibody was used as a negative control, added in Methods, page 8, rows 155-156.

3. **Original question 4. These results are still somewhat perplexing, but they appear to be experimentally sound and sufficiently explained.**

We don`t have anything else to add.

4. **Original question 6. Tables 3 and 4 still have data missing. Please present all multivariate hazard ratios, confidence intervals and p values for all variables assessed in Table 3, and all HRs, CIs and p values in Table 4. There should be no blank spaces in the tables – all data should be presented regardless of significance so readers can transparently assess the results. Statistically significant results can be highlighted with asterisks or bolding of numbers if desired – if so please make sure this is done consistently across all tables.**

All assessed multivariate hazard ratios, confidence intervals and p values are added in Table 3 and 4. Pages 17-18.

Statistically significant p-values are bolded in all Tables.

5. **Original question 7. Thank you for presenting complete data in Table S3. However there still appears to be data missing from Table S2. Please fill in the blanks as in point 6. Also, in table 2, some entries contain two numbers separated by a comma. What is meant by this? Should the comma be a decimal point?**

P-values added in former Table S2, now named Table S3 (changed according to the reference order in the manuscript).

Commas of the entries changed to the decimal points in Table 2, page 12-13.

6. **Original question 8. Please cite the four studies in the text on p4.**

Four studies cited in the manuscript, page 4, row 98. References 12-15.

7. **Original question 9. Please ensure all references in the Supplementary materials and the main manuscript are formatted the same way, consistent with PLoS One’s standards. Some references have authors’ first names while most have only initials.**

References of the manuscript and the Supplementary material are corrected and formatted consistently with PLos One`s standards. Authors first names have removed and changed to initials.

8. **Original question 11. The Discussion regarding omental metastases (e.g. p21 lines 389-406) could benefit from more detail as to how omental metastases occur, if this is known. Do these secondary tumours form as in-transit metastases along blood vessels or lymphatics? Or are they simply the result of local invasion through the parenchyme or along the surface of the omentum, only engaging blood vessels when the deposit becomes large enough to be hypoxic? This also relates back to point 1.**

We have added the principle mechanism of omental metastasis in Discussion, page 22, rows 414-417.

The omental metastasis is the result of implantation of the cancer cells, exfoliated from the primary tumors and spreaded by ascites, to the omental surface and engaging blood vessels when the tumor becomes large enough to be hypoxic.

**Response to Reviewer 4**

We thank Reviewer for the valuable comments. Each of your comment or question is indicated in bold followed by our response. All changes are also highlighted in the “Revised manuscript with track changes” and corrected in the unmarked version of revised manuscript without track changes.

1. **Figure 1 – The median is difficult to see when represented as a larger dot of the same colour as all the other data points. Please use a horizontal line of a different colour to indicate the median IRS. Also, please use grey transparent dots for each data point, to allow readers to distinguish data points that are close together.**

Each single data point is now changed to grey dots to distinguish them from each other and from the black dot describing the median values of the groups in Figure 1.

2. **Throughout the manuscript, the authors refer interchangeably to “high-grade serous ovarian tumors”, and “high grade serous and endometrioid tumors”, and “primary high grade cancer”, and “primary ovarian tumors”. This makes the text difficult to follow. According to Table 1, high-grade serous tumors make up 45 of the 86 ovarian carcinomas tested in this study. If the authors would like to draw specific conclusions about high-grade serous (e.g. lines 201-202), please refer to figures or tables that display results for those specific 45 high-grade serous tumors. Or equally, if the authors would like to make conclusions about high-grade serous and high-grade endometrioid combined (e.g. lines 205-207), please refer to figures or tables in which those 45 high-grade serous have been combined with the 13 high-grade endometrioid tumors, and provide a rationale for doing so. Or if the authors would like to present results pertaining to all ovarian carcinomas combined (e.g. Figure 1 and Table 2), please clearly state this in the text.**

We have standardized the reference of the groups in the manuscript by removing ”high grade serous” and ”high grade serous and endometrioid” groups from the text and pertaining all primary ovarian tumors and refering to the Figure 1 and Table 2.

Pages 10-11, rows 205, 208-209, 232, 236

When comparing related primary and metastatic tumors, the term ”high grade serous” is used, because all 16 metastases were high grade serous as well as obviously their primary tumors. Also the clearest advantage of overall survival was seen in high grade serous group, so that`s why in Table 3 and 4, the high grade serous group is reported separately.

3. **Related to the above issue, throughout the text, many statements in the Results section are still not linked to a Figure or Table. There are too many to list.**

We have added the references to the Tables and Figures. Page 10, rows 208, 210, 212; page 11, rows 231, 233, 235, 237; page 14, rows 260, 268, 269; page 15, rows 278, 285, 288, 292; page 16, rows 303, 305, 306, 310.

To make it more convenient for the readers, we would not want to repeat the references to the Tables and Figures after every sentence.

4. **According to the Methods, ten randomly selected microscopic fields were examined per tumor section. Please include in the Methods a statistical measure on how consistent the scores were between microscopic fields, and state whether the final scores for each tumor were an average of all 10 fields.**

It is not possible to present the numerical values of each single microscopic fields as there were 306 samples with ten microscopic fields each making 3060 calculations. The mean percentage of positive stained cells and intensity was estimated as stated in the Methods, page 8, rows 159-166.

5. **Table 2 – How the p values relate to the data presented in the table is still unclear. If the paired Wilcoxon test was used to compare expression of the paired primary and metastatic high-grade serous tumours (as stated by the authors response to reviewer), the table should show the IRS, PP and SI results for the 16 primary high-grade serous tumours alongside their matched 16 high-grade serous metastases, as that is the data that the Wilcoxon test is being applied to. In addition, all the other unpaired primary tumour IRS, PP and SI results should also be presented in a separate column. Also, Wilcoxon seems appropriate to compare paired IRS and PP scores, but how would that be applied to SI? The SI results are presented in the table as a proportion of tumours that are weak, moderate or strong, so wouldn’t a chi-square test be more appropriate?**

The results of the 16 primary high grade serous tumors are now added to the Table 2 alongside with their matched metastases. The results of the whole study population are left in their own column. Wilcoxon test is used to compare paired IRS and PP values and Pearson`s chi-square test to SI results and that is added to the Table 2 caption, page 12-13.

6. **Table S2 – This table is hard to follow as currently presented. Why is SI reported under Residual tumor, and not the other clinical characteristics, and how is SI reported here as values between 62 and 88, when in the methods it was scored as “0, negative; 1, weak; 2, moderate; 3, strong”? In addition, for some clinical characteristics there appear to be too many p values reported, whilst under Histology the PP Tie-1 p value is missing. Please edit this table to be easier to follow.**

We modified the former Table S2 (now Table S3) according to the suggestions of the previous revision and added the median values of each subgroup in the Table. Now, to make it more readable we took the SI of the residual tumor group away (there were the percentage of the strong staining values reported as 62, 86 and 88, not the exact scores).

Also according to the suggestions of Reviewer 3, all the p-values should be presented, so we added all the p-values, also the values under Histology (and PP Tie-1) groups.

7. **Results, lines 266-268, the authors state that “The low epithelial expression of Ang-2 in primary tumors associated significantly with the ovarian cancer recurrence and with the greater residual tumor size (> 1cm) after primary surgery (p = 0.018, p = 0.012).” However, in Table S2 the median IRS of Ang-2 is 6 both in patients with recurrence and without recurrence, and the median IRS of Ang-2 is also 6 regardless of residual tumor status, which seems to indicate there is no significant difference between any of these groups. This calls into question the conclusions drawn from this part of the analysis.**

We were also wondering that, but as I mentioned in the last revision, according to the statistician, in non-normally distributed groups, the median value can be the same even if there is a significant difference between the groups.

I also calculated this by checking the IRS values of each subgroup and its individual patients. For example in ”no recurrence” group there were 17 patients of total 27 patients (37%), who had low Ang-2 IRS (6 or under) and in ”yes recurrence” group there were 35 patients of total 40 (88%), who had low Ang-2 IRS. And yet the median IRS of both groups was 6. Similarly with the primary recidual tumor, in the ”no recidual” group 28/40 patients (70%) had low Ang-2 IRS and in ”recidual >1cm” group 37/40 (93%) had low Ang-2 IRS.

8. **Figure 3 – Kaplan Meier curves are shown for HGSC patients for Tie-2 (n=45), but not for all ovarian (n=86). Whereas K-M curves are shown for all patients for Ang-2 (n=86), but not HGSC (n=45). Please show the omitted Kaplan-Meier curves.**

Kaplan Meier curves for Tie-2 IRS of all ovarian carcinoma patients and Ang-2 of HGSC group are added to the Figure 3 and Figure caption, page 16, rows 323-324.

9. **If the authors have noted in their correspondence to reviewers that Tie-2 and Ang-2 are correlated in non-HGSC tumours, and do not correlate in HGSC, please state this explicitly in the text of the manuscript for readers, and use the Discussion to explore what this might mean.**

As we stated in the manuscript Tie-2 PP and Ang-2 PP of the whole study population had a weak positive correlation, but corresponding IRSs of the whole group did not reach the significance. When considering the high grade serous group neither IRS or PP of Tie-2 and Ang-2 had significant correlation. This might support the theory of high Ang-2 not being a significant factor for poor OS as we have mentioned in Discussion, page 23, rows 438-439. Otherwise prolonging the Discussion further, as IRS is the main parameter to make conclusions, is not relevant and affects the readability of the manuscript in our opinion. This is also because of the very weak correlation of only PPs.

Added in the Results, page 13, row 251.

10. **Please state in the Methods how the high and low cut-offs were assigned for each biomarker.**

Assignement of cut-off values for each biomarker was made according to median value of the biomarker and this is now stated in Methods, page 8-9, rows 166-169.

11. **Table S3 - The qRT-PCR data needs to be mentioned in the Results, even if the Table was relocated to the Supporting Information. This data does call into question the specificity of the antibodies, or the specificity of the PCR, considering the Ang-2 correlation was significantly negatively correlated, and the Tie-1 and Tie-2 correlations were considerably weak.**

Chapter of qRT-PCR data added to the Results, page 14, rows 265-272.

As mentioned previously the expression of tumor cells was evaluated by immunohistochemistry in this study and PCR method is not able to evaluate the localization of the biomarker. In usual qRT-PCR analysis, different tissues including tumor stroma are mixed.
