# Supplementary material for: High expression of Tie-2 predicts poor prognosis in primary high grade serous ovarian cancer
Source: PLoS One. 2020 Nov 5;15(11):e0241484. doi: 10.1371/journal.pone.0241484 (PMC7644024; doi:10.1371/journal.pone.0241484)
Supplement: Attachment — Submitted filename: Response to Reviewers.docx [file pone.0241484.s007.docx]

A point-by-point Response letter to the Reviewers

**Response to Reviewer 3**

We thank Reviewer for the valuable comments. Each of your comment or question is indicated in bold followed by our response. All changes are also highlighted in the “Revised manuscript with track changes” and corrected in the unmarked version of revised manuscript without track changes.

**Reviewer #3: 1. This section of the Discussion is much improved. However, in acknowledgement that this study does not provide any direct conclusions regarding the relationship between Ang/Tie expression and angiogenesis in ovarian cancer, please delete the word “angiogenic” from the conclusion in line 452, and insert the word “potentially” before “reflecting the more active angiogenesis” on line 449. Possible effects of tumour-expressed Tie2 and Ang2 on pro-angiogenic signalling through endothelial-expressed receptors can be inferred and discussed but they have not been shown directly in this study.**

Deleted word ”angiogenic” in page 23, row 449.

Inserted word ”potentially” in page 23, row 446.

**2. Original Q3: Thank you for the additional detail in the caption for Figure S1, but please be more precise with the descriptions. In the Methods the authors have defined the negative controls as tissues stained with no primary antibody, whereas in the caption the negative controls are described as “negative control tissue”. “Negative control tissue” would imply an image derived by staining a tissue that does not endogenously express a protein with a specific antibody for that protein. Panels b), d) and f) are probably better described as “ovarian carcinoma negative control staining (primary antibody omitted)”. A more stringent standard for IHC controls would be to perform specific antibody staining and negative control staining on serial sections of the same (positively staining) tissue; thus it is not ideal that the Tie1 positive control is tonsil and the negative control is ovarian carcinoma.**

Modified the caption for Figure S1, page 28, rows 623-24.

**3. Original Q4: The fact that IHC and qRT-PCR results for Ang2 correlate negatively in one tissue and positively in another is confusing. The authors may therefore wish to consider removing this information from the abstract.**

Information removed from the Abstract.

**4. Original Q6: It will be important for the authors to check the final formatting of Table 3 by the journal to ensure the results are read correctly and the results and parameter headings are in the correct hierarchy. I would suggest adding a subheading “immunohistochemical staining” above the IRS results and e.g. “clinical features” above stage/ascites and then un-bolding the IRS parameters and stage/ascites to bring these into better alignment with the other sections. This will make it clearer which comparisons relate to overall survival and which to progression free survival when both sets of results are presented within the one table.**

Subheadings added and parameters unbolded in Table 3, page 17-18.

**5. Original Q7: The commas in Tables 2 and S3 appear not to have been changed to decimal points. Please amend. Also the presentation of the data in Table S3 is still confusing. Please clarify in the table caption if the data presented here represent epithelial staining, stromal staining or both. The text (p15 line 285) states that low Tie-1 expression was associated with a greater residual tumour size (>1cm) after primary surgery (p = 0.008), but in Table S3 the p value 0.008 is placed corresponding to zero residual tumour. Similarly I cannot find the stated p = 0.006 corresponding to significantly lower Tie1 expression in high grade tumours, and there are several other examples where the p values cited in the text cannot be found against the corresponding variables in Table S3. Why is this? I assume the remaining blank spaces against a parameter in this table (e.g. for >1 cm residual tumour, Yes recurrence) imply that that parameter was used as the reference for the comparison (with the exception of Histology where there is no obvious reference). Should the 0 residual tumour be the reference condition in Table S3 as it is in Table 3? Please confirm this and ensure that the results are correctly aligned within the table. Also change “0” residual tumour in Table S3 to “None” to be consistent with Table 3. In Table 4 the reference conditions are explicitly labelled but I don’t think this is the norm. Be consistent throughout all tables.**

Commas changed to decimal points in Table 2, page 12-13 and S3.

Modified caption of Table S3, page 28, rows 618-19.

Modified Table S3:

- P-value 0.008 is placed corresponding zero residual tumor.
- P-value 0.006 corresponding significantly lower Tie-1 in high grade tumors is associated only with serous* tumors and when the whole study population (all histologies) is taken into account the corresponding p-value is 0.018 (as marked in Table S3 and said later in the manuscript text, page 15, rows 285-86). We prefered to report the results of the whole population. When the result of the whole study population is not significant, the p-value of subgroup of serous histology is reported, if it is significant (marked with *).
- Consistency with the manuscript text and the Table S3 have been checked.
- The p-values of the Table S3 are changed to the rows of corresponding significant subgroups the values describe.
- Yes, the ”0 residual tumor” is the reference group into which the other groups have been compared to and the results are now aligned correctly within the Table S3.
- The name of ”0 residual tumor” has been changed to ”none residual tumor” in Table S3.

The words ”reference” has been removed in Table 4, page 18.

**6. Original Q8: OK**

We do not have anything to add.

**7. Original Q9: OK**

We do not have anything to add.

**8. Original Q11: Thank you, this part of the discussion is much improved.**

Thank You for the evaluation and review.

**Other comments and Typographical/grammatical errors:
I have roughly proofread the manuscript for English expression. Note that this journal does not provide copyediting, so the authors must please proofread all text, figures and tables very carefully.**

We have proofread all text, figures and tables carefully for English expression.

**Figures and Tables should be referred to as “Figure X” or “Table X” throughout – this appears to only be the wrong way around for the supplementary figures and tables.**

We have changed the writing form of references for supplementary figures and tables.

**P2 line 37: add comma after “ovarian cancer”**

Added comma, page 2, row 37.

**P2 line 39 and line 41: change “expressions” to “expression”, and delete “the” before “angiopoietin-2” on line 39.**

Modified in page 2, rows 39, 41 and 39.

**P3 line 64: add comma after “angiogenesis”**

Added comma, page 3, row 61.

**P3 line 70: There are many endothelial growth factor systems. Sentence should read “Other endothelial growth factor systems, such as the angiopoietin-Tie complex, have not been as extensively studied.”**

Modified, page 3, row 67.

**P3 line 81: replace “speeded up” with “accelerated”; remove “the” before “targeted”**

Modified, page 3, row 78.

**P4 lines 89-90: amend to read “has prolonged median progression free survival (PFS)”**

Amended the expression, page 4, rows 86-87.

**P4 line 100: delete “the” before “dissemination”**

Deleted ”the”, page 4, row 97.

**P4 lines 103, 104: amend “expressions” to “expression**

Amended, page 4, rows 100, 101.

**P8 line 143: delete “the” before “written”**

Deleted ”the”, page 7, row 141.

**p11 line 238: amend to “between expression in”**

Amended, page 11, row 235.

**p14 lines 255-257: section heading could be amended to “Expression of angiogenic factors in primary high grade serous tumours as compared to related metastases”. First sentence could be amended to “Expression of both Ang-2 and Tie-2 …. was”**

Modified the expression, page 13, rows 252-54.

**p14 lines 265-272: the concept of “qRT-PCR levels” doesn’t make sense – qRT-PCR is a technique that measures mRNA levels. I would suggest amending this section to the following:
Correlation of qRT-PCR results with immunohistochemical staining
Ang-2 IRS was significantly negatively correlated to the mRNA levels of Ang-2 measured by qRT-PCR in primary ovarian cancer (r = -0.64, p = 0.002). The correlation was even stronger when only high grade serous tumors were counted (r = -0.868, p < 0.001) (Table S2). In metastatic tumors Ang-2 PP was strongly correlated to the corresponding mRNA levels (r = 0.752, p = 0.005) (Table S2). Tie-1 and Tie-2 qRT-PCR values correlated strongly to each other (r = 0.919, p < 0.001), but did not have statistically significant correlations to the respective immunohistochemical expression. In metastases, mRNA levels of Ang-2 correlated strongly with Tie-2 mRNA (r= 0.61, p= 0.016).**

Modified sentences, page 14, rows 262-69.

**Likewise in the caption:
S2 Table.
Correlation of qRT-PCR results with immunohistochemical staining. Ang-2 IRS and PP were correlated with relative mRNA levels as measured by qRT-PCR. There were 22 primary tumor samples and 15 metastatic samples in each group.**

Modified caption, page 28, rows 613-15.

**P14 lines 276-277: delete “the” before “ovarian” and “greater”**

Deleted ”the”, page 14, rows 273-74.

**P15 line 280: delete “the” before “resistance”**

Deleted ”the”, page 15, row 277.

**P15 line 297: delete “the” before “ovarian”**

Deleted ”the”, page 15, row 294.

**P16 line 324: delete “the” before “significance”**

Deleted ”the”, page 16, row 321.

**P19 line 354: amend “expressed widely” to “widely expressed”**

Amended the expression, page 19, row 351.

**P21 line 390: add comma after “in addition”**

Added comma, page 21, row 387.

**P21 line 398: amend to “Expression of Ang-2 and Tie-2”**

Amended, page 21, row 395.

**P22 line 414-415: delete “the” before “omental”; amend “exfoliating” to “exfoliation”; add “the” before “omental surface”**

Modified, page 22, rows 411-12.

**P22 lines 425-427: delete “the” before “lower” and “larger residual tumour”. Also every instance of “the” could be deleted in the sentence beginning “The expression of Tie-1”.**

Modified, page 22, rows 422-24.

**P23 line 434: amend to “Ang-2 specific andtibody and VEGF blocker aflibercept more strongly reduced tumour growth”**

Modified, page 22, row 431.

**P23 line 453: delete “the” before “OS”.**

Deleted ”the”, page 23, row 450.

**Response to Reviewer 4**

We thank Reviewer for the valuable comment. Your comment or question is indicated in bold followed by our response. All changes are also highlighted in the “Revised manuscript with track changes” and corrected in the unmarked version of revised manuscript without track changes.

**4. Have the authors made all data underlying the findings in their manuscript fully available?**

**Reviewer #4: No**

We think, that all necessery data underlying the findings of the manuscript is written in the manuscript. If additional information concerning the data is desired, it is fully available from the authors upon reasonable request.
