# Supplementary material for: High expression of Tie-2 predicts poor prognosis in primary high grade serous ovarian cancer
Source: PLoS One. 2020 Nov 5;15(11):e0241484. doi: 10.1371/journal.pone.0241484 (PMC7644024; doi:10.1371/journal.pone.0241484)
Supplement: Attachment — Submitted filename: Response to Reviewers.docx [file pone.0241484.s008.docx]

A point-by-point Response letter to the Reviewers

**Response to Reviewer 3**

We thank Reviewer for the valuable comments. Each of your comment or question is indicated in bold followed by our response. All changes are also highlighted in the “Revised manuscript with track changes” and corrected in the unmarked version of revised manuscript without track changes.

**Reviewer #3: All of my previous comments have been satisfactorily addressed. However there are still some problems with Table S3.

Original Q7: a) some of the data in Table S3 still seems to be misaligned. Should all of the p values cited for the Stage analyses be aligned with the III-IV rows rather than the I-II rows? Similarly, should the p values related to Recurrence all be aligned with the Yes rows?**

P-values for the stage analysis are aligned with the III-IV rows and p-values of the recurrence are aligned with the Yes rows in Table S3 of Supporting information.  **b) Thank you for your explanation of the reporting of p values for serous vs total tumours in Table. S3. Please explain this more clearly in the legend, e.g. “* p value reported for serous tumours when only comparison for this subgroup was statistically significant”. However, if this is the case, why are the p values 0.938 and 0.605 corresponding to Tie1 staining with residual tumour <1cm also marked with *? Do these p values represent the whole study population, or serous tumours only? To avoid confusion, please present the all p values (significant or not) for the whole study population in Table S3 to ensure consistency throughout the table. For the instances where the p value was only statistically significant for serous tumours, include this value with * and in brackets after the value for the whole study population; e.g. 0.###(0.008*).**

”p value reported for serous tumours when only comparison for this subgroup was statistically significant” is now explained in the legend of Table S3, page 28, rows 618-19 and under the Table S3 in Supporting information.

P values 0.938 and 0.605 corresponding to Tie1 residual tumor <1cm represent serous tumors when comparing the <1cm group to 0 group, as the only significance was found in serous group (>1cm vs 0). Now we have changed the p values of those particular lines to represent the values of whole study population in Table S3.

We have now presented all p values for the whole study population and when the p value of only serous tumors was significant, it is included in brackets after the value of whole study population in Table S3.

**Response to Reviewer 4**

We thank Reviewer for the valuable comment. Your comment or question is indicated in bold followed by our response. All changes are also highlighted in the “Revised manuscript with track changes” and corrected in the unmarked version of revised manuscript without track changes.

**Reviewer #4: Please carefully review the PLOS Data Policy https://journals.plos.org/plosone/s/data-availability

The following points extracted from the Policy are particularly relevant:

"PLOS journals require authors to make all data necessary to replicate their study’s findings publicly available without restriction at the time of publication. When specific legal or ethical restrictions prohibit public sharing of a data set, authors must indicate how others may obtain access to the data."

"Authors must share the “minimal data set” for their submission. PLOS defines the minimal data set to consist of the data required to replicate all study findings reported in the article, as well as related metadata and methods... For example, authors should submit the following data: The values behind the means, standard deviations and other measures reported;
The values used to build graphs..."

"Stating ‘data available on request from the author’ is not sufficient."**

We have now included the ”minimal data set” in excel format as Supporting information in the submission according to the PLOS Data Policy.
